# Supplementary material for: THO Complex Subunit 7 Homolog Negatively Regulates Cellular Antiviral Response against RNA Viruses by Targeting TBK1
Source: Viruses. 2019 Feb 15;11(2):158. doi: 10.3390/v11020158 (PMC6410154; doi:10.3390/v11020158)
Supplement: Supplementary file 1 [file viruses-11-00158-s001.pdf]

He et al., Fig. S1

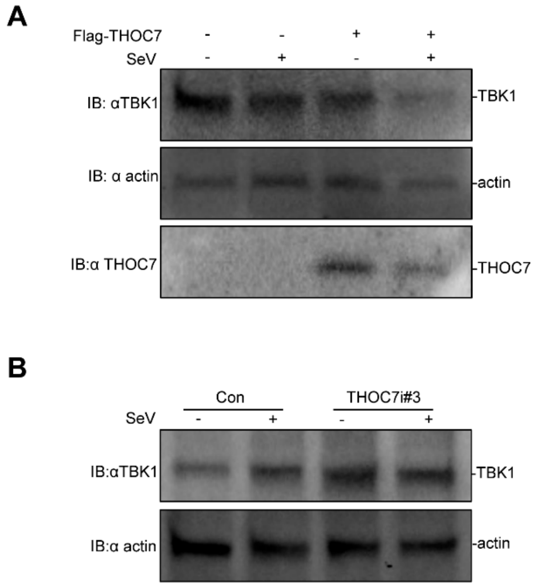

Figure S1. (A) Overexpression of THOC7 promoted degradation of TBK1 in MCF7 cells. MCF7 cells were seeded into 6-well plates and transfected with Flag-THOC7 (3 μg). After transfection for 12 h, the cells were treated with SeV or not for 10 h. (B) Knockdown of THOC7 increased the expression of TBK1 in MCF7 cells. Similar experiments were performed as (A) except transfection with RNAi control and THOC7i#3.
